# The COVID-19 pandemic impact on the Polish medical personnel work: a survey and in-depth interviews study

**DOI:** 10.3389/fpubh.2023.1187312

**Published:** 2023-06-13

**Authors:** Paweł Przyłęcki, Magdalena Wieczorkowska, Agnieszka Pawlak-Kałuzińska, Wioletta Cedrowska-Adamus, Ewa Gulczyńska

**Affiliations:** ^1^Department of Humanistic Sciences, Faculty of Health Sciences, Medical University of Lodz, Łódź, Poland; ^2^Department of Neonatology, Intensive Care and Pathology of Neonate, Institute of the Polish Mother’s Health Center, Łódź, Poland

**Keywords:** healthcare professionals, COVID-19, pandemic, work performance, work overload, stress

## Abstract

**Objective:**

The objective of the study was to examine the impact of the COVID-19 pandemic on the work of medical personnel in terms of: task scope, preparation to perform medical tasks related to the pandemic, team collaboration, involvement in tasks performed, concerns about performing tasks related to the pandemic, stress levels.

**Methods:**

The mixed-method approach was applied to this cross-sectional study. The online questionnaire which included 40 questions was completed via Google among medical personnel in Poland. Eight semi-structured, in-depth interviews were conducted to deepen the data obtained with the questionnaires.

**Participants:**

The questionnaire was completed by 215 healthcare professionals, with the largest group being nurses (56.3%) followed by physicians (22.3%), midwives (11.6%) and other healthcare professionals (e.g., physiotherapists, paramedics, nutritionists – 9.8%). Among the respondents were people who worked in the hospital in the so-called “covid wards” (31.2%) and other hospital wards (60%) as well as people who were employed outside the hospital (8.8%).

**Results:**

The pandemic affected the nature and range of tasks performed by health professionals. Initially, respondents felt unprepared to work under pandemic conditions, but over time their ratings increased in all areas studied. More than half of respondents reported no change in interpersonal relationship within the team, but nearly 35% noted a worsening and only one in 10 claimed improvement. Study participants rated their own commitment to tasks slightly higher than that of their colleagues (mean 4.9 and 4.4 respectively) but the overall rating was high. The mean self-rating of work stress increased from 3.7 before the pandemic to 5.1 during the pandemic. Most of the respondents were afraid of transmission of the infection to their relatives. Other fears included the possibility of making a medical error, not being able to help the patient, not having enough personal protective equipment (PPE) and contracting SARS-CoV-2.

**Conclusion:**

The conducted study revealed that the organization of medical care in the initial period of the pandemic, especially the hospital care of patients infected with SARS-CoV-2, was quite chaotic. The most affected were the people who were transferred to work in the covid wards. Not all medical professionals were prepared to work with the COVID-19 patients, as they lacked experience working in such facilities, especially in intensive care units (ICU). Working under time pressure and under new conditions led mainly to an increase in perceived stress and conflicts between staff.

## Introduction

The first cases of coronavirus disease (COVID-19) caused by severe acute respiratory syndrome coronavirus 2 (SARS-CoV-2) were reported in December 2019 and January 2020 in the Chinese city of Wuhan, Hubei province (1). According to the World Health Organization (WHO), there have been 757,264,511 confirmed cases of COVID-19 worldwide as of February 21, 2023, including 6,850,594 deaths (2). According to the Polish Ministry of Health, the first case of novel coronavirus was confirmed in Poland on March 4, 2020 (3). By February 21, 2023, there were 6,404,036 of confirmed cases, including 118,833 deaths (2).

The rapidly growing number of infected people requiring immediate medical attention in the first weeks of the pandemic threatened the efficiency and resilience of most health systems worldwide (4–6).

From the first days that the novel SARS-CoV-2 virus emerged, HCWs around the world have been on the front lines fighting the unknown disease. Physicians, nurses, paramedics, assistants and medical technicians were the first to take care for patients suffering from this new condition. They had to face the risk of infection and transmission of the virus to their family members. Care had to be provided under new restrictions, without special medications, without equipment for the patients, and sometimes without adequate PPE (7). HCWs had to take on new tasks resulting from the restrictions. These included: wearing protective clothing and frequent disinfection, following complex triage and protective procedures, performing new tasks due to safety restrictions as well as taking on new responsibilities due to relocation to other wards and facilities, performing additional activities alongside patients due to their - usually - serious condition, and increased workload due to increased staff absenteeism. According to WHO, healthcare worker infections accounted for 12.5% of all SARS-CoV-2 infections in the period between March and July 2020 (8). In addition, they had to cope with environmental pressures caused by the high mortality rate of their patients, the fear and anger of patients’ families who could not visit their relatives in the hospital, and the stigmatization of the normal population who viewed health workers as a possible source of infection (9–13).

Previous studies conducted during the Severe Acute Respiratory Syndrome (SARS) outbreak in 2003 and the Middle East Respiratory Syndrome (MERS) outbreak in 2015 reported numerous adverse consequences for healthcare workers. Studies on SARS showed that they had to help more and more patients, even though there was no specific treatment, while preventing them from infecting themselves, which was difficult due to the insufficient amount of PPE. This increased their fear of becoming infected and transmitting the virus to their family members (14–16). Additional tasks they faced included caring for their sick colleagues, which was accompanied by lack of support at work and stigmatization (16, 17).

Similar to SARS and MERS, the SARS-CoV-2 outbreak revealed HCWs were not adequately prepared in terms of equipment, procedures and medications (11). The problem of the shortage of supplies was experienced by medical personnel all over the world, especially in the first weeks of the pandemic. Not only was material support inadequate - health professionals felt unprepared to treat their patients appropriately in terms of standards, procedures and adequate knowledge regarding the virus itself (12, 18, 19). The insecurity in the workplace related to the new tasks mentioned above affected both their physical and mental condition. They reported feelings of insecurity, insomnia, isolation and loneliness, helplessness, alienation, stigmatization, and increased workload and rigid expectations (10, 12, 13, 20–23). In addition, fear of contracting the virus and/or passing it on to family members increased the likelihood of burnout occurrence (24).

Another consequence could be that health workers are reluctant to continue their work under the above circumstances. According to the review conducted in the Bangladesh study, the potential level of absenteeism among health workers during an epidemic ranged from 50% in the UK, 43% in Taiwan, 33% in Australia, 28% in Germany and 16% in Hong Kong, while in Bangladesh 8.9% physicians reported that they were unwilling to work during the first wave of the pandemic. The main barriers and concerns included: fear of transmitting the virus to family members, current health status (co-morbidity), lack of appropriate safety measures, adequate training and working environment, and fear of infection (25).

Undoubtedly, frontline medical workers are at the highest risk of coronavirus infection, which is a real threat to the health care system in most countries of the world. Therefore, high quality PPE and rigorous procedures have been critical to provide an adequate level of safety to those who risk their lives daily caring for infected patients.

Polish HCWs face similar difficulties to their international counterparts, although empirical evidence is scarce (26–32). The available literature either describes their working conditions in the context of recommendations and reviews of implemented government regulations or focuses exclusively on the psychological aspects of working in the pandemic (33, 34).

In addition, much of the international literature focuses on the psychological consequences and deterioration of mental health of frontline health workers, including stress, depression, anxiety, insomnia, PTSD, burnout and depersonalization (10, 13, 35–38), rather than on social aspects such as health workers experiences and evaluation of the following: changes in the work organization, quality of relationships and communication within medical teams and between HCWs and patients, degree of preparation of HCWs for new tasks, perceived commitment of colleagues in performing demanding tasks properly. From this we can conclude that the current state of research still has some gaps that have not been addressed. One of them is the insufficient knowledge about the impact of the pandemic on the social life of HCWs. Previous studies focused mainly on the health and psychological consequences and did not address issues related to the personal lives of health workers. While physicians and nurses seem to be the best-studied medical professions, there is little information on other professional groups (midwives, dentists, assistants). Moreover, many previous studies have listed negative consequences and offered solutions and good practices to be implemented. Therefore, there is an urgent need for follow-up studies that assess the solutions applied to improve the situation of health professionals.

Therefore, our study was designed to answer one of the above questions. The aim of the project was to investigate the impact of the pandemic COVID-19 on the professional and private lives of medical personnel. This article presents partial results on the impact of the pandemic COVID-19 on the work of medical staff in terms of the scope of tasks, preparation for performing medical tasks related to the pandemic, teamwork, involvement in tasks performed, concerns about performing tasks during the pandemic and stress levels. The aim of the study was to investigate the causes and consequences of the changes in tasks and professional activities caused by the pandemic, and to find out how they affected the professional and social relationships of medical staff in the workplace.

## Materials and methods

### The study design

The mixed methods approach was applied to this cross-sectional study. As the study took place during pandemic restrictions, access to hospitals and outpatient clinics was not possible. Therefore, the standardised online questionnaire was conducted among medical personnel in Poland. The elaboration of the questionnaire was done in accordance with the European Statistical System. The first step was to elaborate - during an online meeting of three researchers - the main questions related to the experience of frontline medical staff, their opinion about the virus itself and their assessment of the decisions and strategies implemented to combat the pandemic in Poland. This led to the development of the first version of the questionnaire, which was reviewed by the other two team members, the health workers. Subsequently, the questionnaire was pre-tested among 6 health professionals. This resulted in minor language corrections, the addition of one question and the modification of three other questions. The final version of the questionnaire was created using Microsoft Forms. The questionnaire consisted of 40 questions about the work of medical staff during the COVID-19 pandemic and was divided into three sections. The first section contained questions about work experiences during the first wave of the pandemic. The second section dealt with opinions about the virus itself and the government’s strategies for dealing with it. The final section collected demographic and occupational information about the participants. The study targeted people who worked in health facilities during the COVID-19 period.

The study was conducted from December 2020 to March 2021, i.e., a 9–12 months after the introduction of data on the pandemic. It was the time when vaccination was not yet available (the first vaccination of medical personnel in Poland was on 27 December 2020).

In order to expand some of the information obtained in the survey, a qualitative study was designed. A list of topics was drawn up, which were discussed with the participants in detailed telephone in-depth interviews.

The data collected in the study was checked for completeness, consistency and quality and exported to the statistical package Statistica version 13.3.

Informed consent was obtained from all study participants, both for the online survey and the telephone interviews.

### Research questions

We posed the following research questions that we hoped to answer based on the research conducted:

1. How did the SARS-CoV-2 pandemic affect the tasks and professional activities of HCWs?
  - Did the workload increase? If so, for what reason?
  - Did respondents feel well prepared for the new tasks and activities associated with working in a pandemic?
2. How did the pandemic affect contacts with others?
  - Did the pandemic affect relationships between staff? If so, in what ways?
  - Has there been a change in the way of communicating with the patients? If Yes, how has the nature of communication changed?
3. How did respondents rate their own and their co-workers’ level of involvement in completing tasks during a pandemic?
  - Has the level of work stress changed during the pandemic compared to before the pandemic?

### Sampling and data collecting procedure

As mentioned earlier, our study consisted of a quantitative and a qualitative investigation. Both parts were conducted under the constraints of the pandemic, when it was not possible to contact our participants directly.

#### Quantitative study

Since the electronic questionnaire was sent to different medical facilities across the country, we had no influence on the selection of the sample. The only criterion for participation in the study was an active medical profession. The questionnaire was posted online in closed groups for medical professionals on Facebook and sent by email to medical professionals working in Polish healthcare institutions. We used both convenient and snowball sampling methods to recruit study participants. First, medical professionals known to the researchers were contacted and asked to complete the survey. Then, further contacts were made by the research team and an open call was made to distribute the questionnaire among colleagues.

Ultimately, 215 healthcare workers participated in the study, including 199 (92.6%) women and 16 (7.4%) men. The study group included 48 (22.3%) physicians, 121 (56.3%) nurses, 25 (11.6%) midwives and 21 (9.8%) workers with another medical profession, such as physiotherapists, paramedics, nutritionists (Table 1). In this group, the vast majority of individuals were employed in a hospital (91.2%), including 67 (31.2%) in the so-called “covid wards” (only for patients infected with SARS-CoV-2 virus) and 129 (60%) in different hospital wards (with patients not suffering from coronavirus). The remaining 19 respondents (8.8%) were employed in various medical facilities outside the hospital.

**Table 1:** Research group (*N* = 215).

Age	Total	Physician (*N* = 48; 22.3%)		Nurse (*N* = 121; 56.3%)		Midwife (*N* = 25; 11.6%)		Other paramedics (*N* = 21; 9.8%)	
		Female (*N* = 38)	Male (*N* = 10)	Female (*N* = 93)	Male (*N* = 28)	Female (*N* = 25)	Male (*N* = 0)	Female (*N* = 21)	Male (*N* = 0)
Work in the hospital, covid ward (*N* = 67; 31.2%)									
18–29	**15**			8	3	4			
30–39	**12**	5	1	6					
40–49	**15**	5	2	5		1		2	
50–59	**20**	2	1	14		1		2	
60 and more	**5**		1	4					
Work in the hospital, non-covid ward (*N* = 129; 60.0%)									
18–29	**41**	2	1	22		14		2	
30–39	**21**	8	1	7	2	1		2	
40–49	**24**	2		12		4		6	
50–59	**33**	4	2	1	23			3	
60 and more	**10**	7		3					
Work outside hospital (*N* = 19; 8.8%)									
18–29	**3**			3					
30–39	**4**		1	1				2	
40–49	**5**	2		2				1	
50–59	**6**	1		4				1	
60 and more	**1**			1					

#### Qualitative study

Eight semi-structured in-depth interviews were conducted to expand on the data collected in the questionnaires. As we wanted to obtain more information mainly from people working in the so-called covid wards (special *ad hoc* hospital wards exclusively treating individuals with SARS-CoV-2), these interviews were conducted with these professionals. This group included only nurses, which was related to the fact that this group was easy to reach and willing to participate in the study. Due to the limitations of COVID-19, it was not possible to conduct face-to-face interviews, so these were telephone interviews. The information obtained was checked for completeness and consistency and then coded and analysed to produce a descriptive elaboration. In-depth individual interviews were conducted with 8 nurses. All of them had experience of working in the covid ward, even though they had worked on this ward at different times during the pandemic. These nurses were employed in three different hospitals. They were recruited using a snowballing process.

### Analysis

#### Statistical analysis

All statistical analyses were performed with the package Statistica version 13.3. The frequency distribution was calculated for categorical variables and the mean with standard deviation was estimated for continuous variables (assessment of stress level, assessment of task performance). In addition, the frequency distribution was used to illustrate: a/ the nature and extent of changes related to new medical tasks performed by healthcare professionals during the COID-19 pandemic, b/ anxiety related to job performance accompanying healthcare professionals, c/ assessment of teamwork. Tests to check for the occurrence of correlations between two variables were conducted using Pearson’s χ2 test. Our independent variables were workplace and position. Dependent variables included changes in job duties during the pandemic and the type of anxiety reported by medical staff. If the correlation was statistically significant, the strength of the relationship was estimated using the V-Cramer’s Ratio. The statistical significance level was set at the value of *p* < 0.05.

#### Qualitative analysis

In order to obtain clear and orderly information, problem analysis was used to elaborate the material obtained in the in-depth interviews. First, the transcription of the interviews was carried out. Then selected themes/threads were distinguished and the material was divided into appropriate sections. Then the problem analysis was carried out to find similarities or/and differences in the statements and opinions of the interviewees.

## Results

The sudden increase in the occurrence of SARS-CoV-2 led to changes in the organization of medical care from top to bottom, the main goal of which was to separate healthy people from those infected with the SARS-CoV-2 virus to prevent the transmission of the virus. As a result, some hospital departments were converted into covid hospitals or covid departments. At the same time, the number of admissions of patients suffering from other diseases was significantly reduced, and those admitted for elective procedures were required to undergo testing to confirm there was no current SARS-CoV-2 infection. In addition, some medical staff were delegated to the newly established covid wards. In ambulatory care, the possibility of telecare was introduced, which had not been used in Poland before.

The COVID-19 pandemic proved to be a challenge for all social and professional groups, and the professional group that was at the forefront of combating the pandemic was the medical staff. This study shows how HCWs managed their professional tasks under the specific conditions of the epidemic, how the nature of their work changed, and the impact of the pandemic on communication between workers during the pandemic.

The results of the study are presented separately. In the first part, the results of a survey are presented in which medical staff in different positions and in different medical facilities participated, both in hospital (covid and non-covid wards) and in outpatient facilities. In the second part, the main problems related to work during the pandemic, especially in the covid wards, are highlighted based on in-depth interviews with nurses.

### Survey results

#### Extent and nature of changes in the job duties

The introduction of the state of the pandemic led to restructuring of health care work. Therefore, we asked respondents to what extent and how the scope of their job duties changed. For the most part, respondents agreed that the pandemic contributed to a change in the scope of their job duties, with 28.8% of respondents indicating that it was a complete change and 56.7% indicating that it was a partial change. The change in the scope of duties performed affected all professional groups. It was found that there was a statistical relationship between the place where the respondents worked (hospital-covid ward, hospital-non covid ward, outpatient clinic) and the change in their job responsibilities (*χ*^2^ = 16.649, *p* = 0.002, *V* = 0.197). However, there is no statistical correlation between the variables “position” and “change in job duties” (Table 2).

**Table 2:** The scope change in the professional tasks performed (*N* = 215).

Change in professional duties			
	Total (*N* = 62; 28.8%)	Partial (*N* = 122; 56.7%)	Small or none (*N* = 31; 14.5%)
Workplace (*N* = 215)			
Hospital, covid work (*N* = 67)	27	33	7
Hospital, non-covid work (*N* = 129)	26	84	19
Outside hospital (*N* = 19)	9	5	5
*X*^2^ *= 16.649; V = 0.197; p = 0.002*			
Position (*N* = 215)			
Physician (*N* = 48)	12	31	5
Nurse (*N* = 121)	35	65	21
Midwife (*N* = 25)	6	17	2
Other (*N* = 21)	9	9	3
*NS*			

The change in the scope of duties was mainly related to the type of communication with patients and medical staff (74%), increased workload mainly due to the need to substitute for sick colleagues at work (69.8%), spending extra time wearing protective clothing (68.4%). More than half of the respondents also reported that the change involved having to perform new responsibilities (60.9%) and having to complete additional documents (60.5%) (Table 3).

**Table 3:** The most common changes in the performance of work during a pandemic (Many answers possible, *N* = 215).

	Extended working hours	work in a different ward	New responsibilities	Necessity to fill in additional documents	More things to do next to the patient	A different way of communication with the patient/family/staff	Greater workload related to increased absenteeism (due to isolation, quarantaine)	Greater workload due to the need to wear clothing in a time-consuming manner	Limiting the possibility of working outside the primary workplace	The need to deal with media hate related to top-down restrictions on visiting patients
	*N* = 33; 15.3%	*N* = 41; 19.1%	*N* = 131; 60.9%	*N* = 130; 60.5%	*N* = 93; 43.3%	*N* = 159; 74.0%	*N* = 150; 69.8%	*N* = 147; 68.4%	*N* = 54; 25.1%	*N* = 80; 37.2%
Position										
Physician (*N* = 48)	8	7	31	34	23	39	31	29	11	14
Nurse (*N* = 121)	21	25	73	69	55	86	87	92	31	48
Midwife (*N* = 25)	3	6	16	18	10	21	18	13	6	13
Other paramedics (*N* = 21)	1	3	11	9	5	13	14	13	6	5
	*NS*	*NS*	*NS*	*NS*	*NS*	*NS*	*NS*	*x*^2^ *= 8.194; V = 0.195; p = 0.042*	*NS*	*NS*
Workplace										
Hospital, covid ward (*N* = 67)	15	26	46	33	28	54	49	52	16	22
Hospital, non-covid ward (*N* = 129)	13	13	71	85	59	94	91	86	32	53
Outside hospital (*N* = 19)	5	2	14	12	6	11	10	9	6	5
	*x*^2^ *= 7.073; V = 0.181; p = 0.029*	*x*^2^ *= 24.567; V = 0.338; p < 0.001*	*NS*	*NS*	*NS*	*NS*	*NS*	*x*^2^ *= 6.695; V = 0.176; p = 0.035*	*NS*	*NS*

To test whether there was a statistically significant correlation between the independent variables such as “position” or “workplace” (hospital – covid ward, hospital – non-covid ward, non-ambulatory medical facility) and the dependent variables in relation to the assessment of changes in the performance of professional duties during the pandemic, Pearson’s chi-square test for independence was used. It was found that there is a statistical dependence between the variables “position” and “greater workload due to time-consuming wearing of clothes” (*χ*^2^ = 8.194, *p* < 0.05, *V* = 0.176). There is also a statistical relationship between the independent variable “workplace” with three variables: a) “extended working hours” (*χ*^2^ = 7.073, *p* < 0.05, *V* = 0.181); b) “working in another ward” (*χ*^2^ = 24.567, *p* < 0.001, *V* = 0.338); c) “greater workload due to time-consuming wearing of clothes *χ*^2^ = 6.695, *p* < 0.05, *V* = 0.176) (Table 3).

#### Preparation for occupational tasks during the COVID-19 pandemic

Respondents were asked to indicate how they rated their preparation for work during a pandemic, both in terms of knowledge and technical equipment. They provided this rating for the first 3 months of the pandemic and at the time of the study, which was period from 9 to 12 month after the pandemic state was declared. Rating was made on a scale of 1 to 4, with 1 indicating definitely bad and 4 definitely good. From the data obtained, it can be concluded that respondents evaluated their individual aspects of work preparation worse at the beginning of the pandemic, and better at the time of the study, which is during the period of 9–12 months after the pandemic was announced. This suggests that over time respondents became accustomed to the pandemic and adapted to working under pandemic conditions, and assessed the access to technical equipment better (Table 4). This is evidenced by the following data:

- evaluation of their own knowledge and skills (increase in mean from 2.4 to 3.3);
- evaluation of the work of teammates (increase in mean from 2.7 to 3.2);
- evaluation of the equipment of the medical facilities with protective measures (increase in mean from 2.4 to 3.2);
- evaluation of medical facilities in terms of access to medical equipment (increase in mean from 2.5 to 3.2).
- evaluation of procedures that allow for proper performance of professional duties (increase in mean from 2.4 to 3.1).

**Table 4:** Self-assessment of the respondents regarding preparation to perform medical tasks during the COVID-19 pandemic (*N* = 215).

	Period*	1	2	3	4	*X̅*	SD
I have the appropriate knowledge and competences to perform medical tasks (*N* = 215)	I	43	77	73	22	2.3	0.913
	II	2	13	128	72	3.3	0.607
I have appropriate personal protective equipment to perform medical tasks (*N* = 215)	I	53	61	70	31	2.4	1.009
	II	8	33	91	83	3.2	0.816
I have the appropriate equipment to perform medical tasks (*N* = 215)	I	41	63	81	30	2.5	0.956
	II	5	27	107	76	3.2	0.736
There are proper procedures that allow me to perform medical tasks (*N* = 214)	I	43	84	55	32	2.4	0.967
	II	4	29	117	65	3.1	0.736
I work with a competent team, thanks to which I can perform medical tasks properly (*N* = 215)	I	24	54	99	38	2.7	0.889
	II	7	20	111	77	3.2	0.738

*I – respondents’ self-assessment of preparedness to practice medical duties during the first 3 months of the pandemic, II – respondents’ self-assessment of preparedness to practice medical duties in the period of 9–12 months after the announcement of the pandemic.

#### Commitment to work

We also wanted to examine how respondents evaluated their own and their colleagues’ commitment to work during a pandemic. Respondents rated themselves on a scale of 1 to 6, with 1 indicating very low commitment and 6 indicating very high commitment to performing medical tasks (Table 5). From the results, it can be concluded that respondents assessed their commitment higher (mean 4.9) than that of their colleagues at work (mean 4.4). However, the difference in assessment was not very large and the overall rating of commitment was high.

**Table 5:** Commitment to performing medical tasks in connection with the COVID-19 pandemic (*N* = 215).

	1	2	3	4	5	6	*X̅*	SD
Assessment of colleagues by the respondents	–	10	26	72	77	30	4.4	1.024
Self-assessment	–	–	15	38	106	56	4.9	0.846

#### Team communication and collaboration

We also examined team relationships related to work during the COVID-19 pandemic. Most respondents reported that relationships had not changed (53.5%), while for some people these relationships had either improved (11.6%) or deteriorated (34.9%) (Table 6). Based on the statistical analysis, it is found that there was no statistically significant relationship between the independent variables “position,” “workplace” and the rating of interpersonal relations in the team.

**Table 6:** Team collaboration (*N* = 215).

	Team interpersonal relationships deteriorated	Team interpersonal relationships improved	Team interpersonal relationships have not changed
	*N* = 75 (34.9%)	*N* = 25 (11.6%)	*N* = 115 (53.5%)
Position	75	25	115
Physician (N = 48)	13	4	31
Nurse (N = 121)	43	18	60
Midwife (N = 25)	8	0	17
Other paramedics (N = 21)	11	3	7
*NS*			
Workplace			
Hospital, covid ward (*N* = 67)	26	10	31
Hospital, non-covid ward (*N* = 129)	41	12	76
Outside hospital (*N* = 19)	8	3	8
*NS*			

#### Stress at work

New situations in the workplace, such as the change in the nature of work that has occurred in relation to the introduction of the pandemic, generally cause stress among workers. Respondents were therefore asked to rate the stress associated with their work in the pre-pandemic and pandemic periods. As expected, the average self-assessment of stress at work among respondents increased from 3.7 to 5.1 (Table 7).

**Table 7:** Self-assessment of stress at work (*N* = 215).

	1	2	3	4	5	6	*X̅*	SD
Before the pandemic	9	23	63	64	40	16	3.7	1.225
During the pandemic	2	2	8	33	91	79	5.1	0.949

There are many reasons for the increase in stress levels at work. One might assume that the main reason is fear for one’s health related to the risk of infection with the SARS-CoV-2 virus. However, it was not the fear of contracting SARS-CoV-2 that was the most important cause of stress for respondents (39.1% of responses), but the fear of the possibility of passing on the infection to their relatives (79.1%). Respondents also pointed to other fears, which mainly include: the possibility of making a medical mistake because they are performing activities beyond their competence (51.2%) or because they are exhausted (47.4%), afraid of being unable to help the patient (47.4%). Interestingly, quite a large group of respondents, 40.5%, were afraid of not having enough PPE. Most surprisingly a small proportion of respondents feared death, both their own (19.1%) and the patient’s (21.4%). As shown in Table 8, a statistical relationship was found between the independent variable “position” and two dependent variables: a) “I am afraid that I will not be able to help the patient because of the excess of responsibility” (*χ*^2^ = 17.028, *p* = 0.001, *V* = 0.281), b) “I am afraid that I may make a mistake of exhaustion” (*χ*^2^ = 12.506, *p* = 0.006, *V* = 0.241). In addition, there is also a statistical correlation between the variables “workplace” and “I am afraid of getting infected” (*χ*^2^ = 6.160, *p* = 0.046, *V* = 0.169).

**Table 8:** Concerns about performing work during a pandemic (Many answers possible) (*N* = 215).

	I am afraid of getting infected	I am afraid that I will infect my loved ones	I am afraid that I will not be able to help the patient due to the lack of equipment	I am afraid that I will not be able to help the patient due to the excess of responsibilities	I am afraid that I will be responsible for mistakes made in connection with the performance of work beyond my competence	I am afraid that I may make a mistake of exhaustion	I’m afraid I will not have personal protective equipment	I’m afraid of the patient’s death	I’m not afraid of anything
Total	*N* = 84; 39.1%	*N* = 170; 79.1%	*N* = 59; 27.4%	*N* = 102; 47.4%	*N* = 110; 51.2%	*N* = 102; 47.4%	*N* = 87; 40.5%	*N* = 46; 21.4%	*N* = 41; 19.1%
Position									
Physician (*N* = 48)	18	37	13	15	23	15	18	15	8
Nurse (*N* = 121)	48	101	36	69	65	69	50	23	26
Midwife (*N* = 25)	8	17	9	14	16	12	12	7	4
Other paramedics (*N* = 21)	10	15	1	4	6	6	7	1	3
	*NS*	*NS*	*NS*	*X*^2^ *= 17.028; V = 0.281; p = 0.001*	*NS*	*X*^2^ *= 12.506; V = 0.241; p = 0.006*	*NS*	*NS*	*NS*
Workplace									
Hospital, covid ward (*N* = 67)	18	45	18	38	36	37	18	12	11
Hospital, non-covid ward (*N* = 129)	57	108	39	58	66	57	61	32	26
Outside hospital (*N* = 19)	9	17	2	6	8	8	8	2	4
	*X*^2^ *= 6.160; V = 0.169; p = 0.046*	*NS*	*NS*	*NS*	*NS*	*NS*	*NS*	*NS*	*NS*

### In-depth interviews

In-depth interviews were conducted only with nurses working in the hospital. It should therefore be emphasised that they describe their view on issues related to the difficulties associated with performing work in a hospital during the pandemic. Thus, these data cannot be considered representative of the group studied, however the information obtained allows us to highlight the nature of the difficulties had encountered by nurses during the pandemic. The analysis of the interviews made it possible to identify several such key problems, which will be discussed below. The statements focus mainly on working in the covid ward, as all the interviewees had more or less experience in working in the covid ward. The respondents’ statements are quoted in their original form. After each is information about the participant, where “I” is an abbreviation of “interviewee” and the numbers describe the subsequent participants in the qualitative study.

#### Delegating to work in the covid ward

According to the in-depth interviews conducted, both doctors and nurses, who often had different professional experiences beforehand, were delegated to work in the covid wards. People work were delegated to work in the covid ward could not refuse to work in this ward. Working in the covid ward was very stressful for the medical staff due to many factors, which we will discuss in more detail later. However, it should be noted that working in the covid ward was particularly demanding and stressful for those who had never worked in the ICU before. There were many such people and they were transferred from above from different hospital departments, such as dermatology or psychiatry.

There were many people who were delegated by the Voivodeship Marshal from other hospitals, other departments, who never had anything to do with the covid, who did not work in the intensive care unit, who did not have to deal with a ventilator, and these people were members of our team, for example. Because there was no recruitment here, but these people were sent to us from above. And these people also had to acclimatize and were confronted with reality, they had to learn everything quickly. At the same time, not everyone had the patience to teach these new people (I_6).

#### The specifics of working in the covid ward

Respondents pointed out that the work and safety measures in the covid ward were different from those in other hospital wards. Although special precautions were taken in hospitals during the pandemic, these were much more restrictive in the covid ward and concerned work organisation, clothing, rules for movement in the ward, division into so-called clean zones (places where it is not necessary to wear tight uniforms because the risk of coronavirus infection is possible but not high) and dirty zones (places such as the sickroom where there is a high risk of coronavirus infection and special precautions must be taken). Working in the covid ward was very difficult for people, especially in the beginning, simply because of the different nature of the work. As one of the people said:

Everything has changed. We are not at all where we were before, in terms of a workplace; now there is a clean area and a dirty area. The clean area is where we change, where we eat, where we wait for patients, for a telephone call, and the dirty area is the one with the coronavirus (I_2).

Respondents pointed to the unprecedented rules on the use of protective clothing, which were also more restrictive than in non-covid wards. The use of appropriate protective measures was time-consuming and, above all, a heavy physical burden for the people who had to spend several hours a day inside.

It is more difficult to perform manual activities because we are dressed differently and your vision is limited by the goggles, which evaporate. Also, we wear three pairs of gloves, whereas before we had one pair of thinner gloves, and here are two pairs of thick gloves and one is thinner, so these manual difficulties are the most difficult. It is this limitation of mobility and field of vision that makes it difficult (I_4).

We cannot endure more than 3–4 h in these clothes. You cannot pee, drink, do physiological stuff, even if you have to go to the pee. When we are there for 6 h, it is unreal, it is artificial, you sweat, it is like being in a treadmill (I_2).

#### Lack of training for new medical equipment

In the studies, respondents frequently raised the thread of the preparation of medical staff assigned to care for patients infected with SARS-CoV-2 in the covid ward. These patients were usually in a very poor condition, unconscious, unable to breathe on their own and had to be put on a ventilator. Some of the staff assigned to work in this ward, both doctors and nurses, were not prepared to take on appropriate medical tasks, especially because they had not worked in the ICU before.

In the covid wards, knowledge and skills in anaesthesia and intensive care were particularly important. The patients on these wards, who were usually unconscious, had to be artificially ventilated to support their breathing. Therefore, it was particularly important to have the ability to operate special medical equipment such as ventilators. However, the respondents stated that no one had tested their knowledge and skills before. These people, and this was especially true for nurses, were thrown in at the deep end. On the first day, no one instructed them on how to use the new medical equipment. In general, the situation was such that the team consisted of different people, both those who had previously worked in the ICU and knew how to operate medical equipment and those who did not. The nurses who participated in the study indicated that those who did not have the appropriate knowledge and skills learned from the more experienced nurses by observing them. These situations sometimes led to more serious conflicts. This was because not all of the more experienced people wanted to explain the rules of using a particular piece of medical equipment to the less experienced nurses. Such a situation occurred especially when new staff joined the team and became tiring for the experienced staff.

In fact, we all learned everything from scratch, so to speak. Organizationally, we were not prepared (…) after all, the chaos and disorder was great at the beginning (I_6).

There was no management and no preparation. I think the state provided a lot of money to build for example, a new temporary hospital, but it did not think about the staff, about the fact that for some people there should even be a two-day training - listen, you are going to be confronted with this and that, you need to know this and that, we are going to put on suits anyway, we should all be trained somehow for certain things, there will be respirators there, we’ll operate them there. There was nothing like that, that was clear (I_6).

There was no training, even with new monitors, kidney dialysis, everything you guessed, seen from the side, then operated. It is also simply unacceptable that there is no training. Kidney training consisted of watching my colleagues and repeating it (I_5).

#### Unequal division of labour between doctors and nurses

Another conflict highlighted by the nurses interviewed concerned the division of labour between physicians and nurses. According to the interviewees, the main burden of contact with the patient laid with the nurse, who spent several hours in a protective suit in the so-called dirty zone, i.e., where one could become infected with the SARS-CoV-2 virus. The doctors, on the other hand, rarely entered this zone where SARS-CoV-2 patients connected to respirators were staying and where protective clothing had to be worn. They were separated from the dirty zone and the nurses. The doctors made recommendations from their office on how to deal with it, sometimes even by phone. Meanwhile, the nurses did all the activities with the patient, including connecting them to the medical equipment. This situation placed an additional burden on them, was stressful and at the same time gave them the feeling of an unfair division of tasks between the doctor and the nurses. As we did not interview doctors, we are aware that the following opinions only reflect the views of nurses.

The doctor enters the ward only when the patient is admitted, places an intra-arterial puncture and connects him to a ventilator. Also, the doctors do not come in but give us instructions over the phone. When something like this happens on the cito, the nurses have to decide for themselves whether to decrease or increase the dose of the drug or whether to insert or remove the tube. And that is exhausting, it should not be like that (I_8).

The doctor was on duty from his room, and there was supervision and he was observing. The nurses were still inside and the doctors came in when it was a round or an emergency, an admission or a resuscitation, got dressed and went in. But it was not like they changed like we did and they were still in the covid ward (I_7).

There was such a lack of interest. Because I think when there are so many doctors on duty and none of them attend the so-called ward rounds or check the depth of the endotracheal tubes. I just think that they keep their activities to a minimum and ours to a maximum, to the extent that they let us dictate the parameters of the ventilators by phone, where the maintenance of the ventilators is absolutely the responsibility of the doctors (…). It happened that we had to leave the area with the patient because 4 h had passed, and for 4 h we were protected because this was the protective material of our clothes, and then the doctor called us and extended this time by 20 min and asked us to dictate exactly these parameters of the ventilator (I_5).

#### Special, emotionally difficult contact with the patient

The in-depth interviews shed further light on the possibility of stressful situations in the workplace, especially in covid wards. The interviewees pointed out that their work in this ward has completely changed due to contact with a very different group of patients than before. Regardless of which ward the medics worked in before, they had more or less contact with the patients. In the covid wards, however, there is almost no such contact with the patients, because almost all patients are unconscious and connected to a ventilator. And even if the patient is conscious, it is almost impossible to communicate with them. Medical staff wear tight uniforms and masks that make conversation difficult. In addition, according to the interviewees, people in the covid wards feel depressed, which is due to both the lack of contact with the patient and the very high mortality rate.

We are shocked by the atmosphere in these covid wards, which is one of depression, fear and loneliness. You really feel a great loneliness in these patients and that is the worst thing (I_1).

There is such a depressive and anxious atmosphere in these wards. These people lie so scared in these beds, they are helpless, there is no family with them, they cannot move like they used to. They are waiting for someone to come to them, even the staff. We go to these patients like robots in these suits. And that is so sad for these people. It shows that others are not smiling, they are depressed (I_3).

In our ward where I work, the mortality is 99%. When we admit a patient to the ward, we know that they will not come out again, and it is depressing that it is only a matter of time. This is a special situation. Because until now we worked in a ward where there were also deaths, but most of the patients went home, and here it is the other way round (I_4).

The interviewees also emphasised that working in the covid ward has a great impact on them. They feel bad when they see the poor condition or mood of the patients.

When the patients see us, they call us. You can see that the patients are sad, they are just sad. I admit that I did not expect that and that makes the biggest impression on me, that these people are sad and depressed (I_1).

Working in the covid ward has also changed the nature of contact between the patient’s family and the patient. Indeed, the impossibility of visiting the hospital was an additional burden on the medical staff who acted as intermediaries in this communication. Such a situation was stressful both in terms of time and, above all, psychologically. The situation, which was a heavy burden for the medical staff, is very well illustrated by the statement of one of the persons:

Recently a family gave a letter to a loved one who was unconscious and asked us to read it out. These are the things that often affect us too, we are not machines that read such things (…). It was the family’s request. And that unfortunately transfers to us, because each of us thought, if he was in such a situation, how would he feel … to be on the other side and not be able to see his loved one. It is common knowledge that it is the mother, the daughter, the grandfather, the grandmother. Apart from the fact that we do all these things, there is also an emotional aspect (…). In the past, it was possible to give a telephone number, even if the patient did not have one of his own. And the sick person was already talking to himself. We nurses tried to go out so as not to embarrass the patient with such a conversation with someone close to him. Now the families here very often ask to call a priest to whisper to the sick person that they love him, that they are waiting for him (I_4).

The results of both the quantitative and qualitative research presented above show that work during the pandemic was stressful for medical staff, and the reason for this was the major changes in the nature of the tasks performed. According to the survey, the extent of the changes was very large and affected different aspects of the work. Respondents indicated in the survey that the most noticeable change for them (74% of responses) was the change in the way they communicated with patients. Specific aspects of this change were mentioned by respondents in the in-depth interviews. It should also be emphasised that working under pandemic conditions significantly affected the physical and mental well-being of medical staff. Medical staff had to wear tight uniforms and protective clothing for many hours, which limited their ability to move freely, speak and perform medical activities. Many medical staff who were assigned to work in covid-wards were not prepared to perform medical tasks, they were thrown into the so-called deep water. In addition, medical workers were under constant stress at work, compounded by fear of infection and especially the possibility of transmitting the virus to their relatives (79.1% of responses). What added to the stress among the health workers were the newly created conflicts between the health workers themselves, largely caused by the chaos and lack of proper management in the first months of the pandemic. Here, both the lack of a clear division of tasks between doctors and nurses and the delegation of people to work in covid wards without prior training, for example in handling medical equipment, should be mentioned.

## Discussion

In our study we posed three main research questions to which we wanted to obtain answers based on the research conducted.

### The impact of the SARS-CoV-2 pandemic on the tasks and professional activities of health workers

The first question related to changes in the tasks and professional activities of health workers due to SARS-CoV-2 pandemic. We wanted to investigate whether the workload had increased and, if so, what the causes were. We were also interested in how health workers perceived their preparation for the new tasks as a result of the pandemic. The pandemic forced medical facilities to introduce new solutions that could initially lead to disorder and chaos. Frontline staff were most affected by the new procedures and tasks, which were also carried out in a reorganised environment and using additional equipment. Most of our respondents, representing all occupational groups, confirmed that the pandemic had contributed to the change in scope of their duties. The place of work (covid or non-covid ward or outpatient clinic) had a significant impact on the change in professional tasks. The study found that the pandemic particularly affected communication with patients and medical staff (74%), and wearing a protective suit made this more difficult. A 2019 study (39) shows that wearing PPE can have a negative impact on healthcare communication.

Our respondents also reported a higher workload due to the need to cover for their sick colleagues (69.8%). According to the 2020 report (40), covering the period from the first case in March to 14 September 2020, approximately 4,000 of all infected Poles were healthcare workers (representing approximately 17% of the total number of COVID-19 patients). The highest infection rates were among nurses (2,393 cases), doctors (986) and midwives (1644). Many of them had to be hospitalised (398, 194 and 27, respectively). In addition, a large number of medical personnel were quarantined (31,077 HCWs), including 18,495 nurses, 8,881 doctors and 1,644 midwives. There were 13 deaths (7 of doctors and 6 of nurses). The Bangladeshi doctors’ study sheds new light on the absenteeism of health workers - according to the authors, almost 9% of respondents were unwilling to work during the pandemic for a variety of reasons (including their health status, fear of infection and fear of transmitting the virus to their relatives) (25). Staff shortages seem to be a major problem during the pandemic. This problem was also mentioned by the nurses interviewed in connection with the transfer of the HCWs to other wards.

Another reported change in duties was the time taken to put on and take off the protective suit (68.4%) and it correlated with the position and workplace. The qualitative study confirmed that these were nurses who were constantly on duty and therefore had to spend more time putting on and taking off protective gear, which was time-consuming. Working in the covid ward also required additional time for putting on and off PPE.

Other changes included taking on new duties (60.9%), completing more documents (60.5%) and more tasks alongside the patient (43.3%). Almost one in five respondents had experienced a move to another ward, which involved new responsibilities and tasks. The transfer to the covid ward could not be refused and was very stressful for the workers who had different professional experiences beforehand. In-depth interviews revealed that it was a great hardship for people with no previous experience of working in the ICU. These problems were especially highlighted in the interviews with the nurses. They described in detail the specifics of the ICU and emphasised the lack of training in using the new equipment. Furthermore, respondents and interviewees admitted that they worked under chaotic organizational conditions, lack of training and a lack of PPE. This confirms the findings from the Bangladesh study (19) which found higher workload and a lack of coordination and quality management. Another qualitative study (41) showed similar changes in tasks in a broader, organizational perspective. Respondents reported difficulties in performing their tasks because they had to change their protective suits frequently. They also felt they no longer had control over their schedules and responsibilities. In addition, a meta-analysis of 161 qualitative studies (4) found that medical staff faced increased workload and new workplace demands.

Our respondents also reported that they had to deal with media hatred related to restrictions on visiting patients imposed from above (37.1%). This is similar to the study from Bangladesh, were HCWs reported social exclusion and stigmatisation (19). Moreover, Donna McKay with the team reports in their paper acts of physical violence against HCWs (42).

At the beginning of the pandemic, participants in our study did not feel well prepared for the new tasks and activities associated with working in new circumstances, but this improved over time. The greatest improvement in scores in the assessment of their own knowledge and skills (from 2.3 to 3.3) and the least in the assessment of teammates’ work (from 2.7 to 3.2), but this score was initially the highest of all four dimensions assessed on scales. Initially, health workers had the most difficulties with lack of knowledge and skills, lack of appropriate procedures, and lack of protective and medical supplies. This is consistent with the findings of previously cited studies (7, 19, 41), but analyses of international and Polish literature allow to suggest that these are global challenges (9, 31, 43–45).

### Impact of the pandemic on contacts with others

Our second question was about the impact of the pandemic on contacts with others. We wanted to know how the pandemic affected relationships between staff and whether it changed the way they communicated with patients. The study found that the pandemic changed the nature of communication with patients and medical staff (74%) and that wearing a protective suit made this more difficult. This fact is confirmed by the study by Hampton et al. (39).

The change in communication was also associated with new tasks and responsibilities, which in turn affected the general relationships between medical staff. Although more than half of the respondents reported no changes in staff relations, almost 35% saw deterioration and only one in 10 claimed that they had improved. In the in-depth interviews, we were able to identify the main reasons for the deterioration in relationships. The first reason was the delegation of people who were not prepared to take on appropriate medical tasks, which led to conflicts about the transfer of qualified knowledge as well as about the quality of the performance of inexperienced medical staff. The second reason concerned the division of labour between doctors and nurses. The latter felt disproportionately burdened compared to the former. In addition, nurses were more exposed to the virus as they performed most of the operations with patients. This is consistent with the findings of a systematic review and qualitative meta synthesis conducted by Billings ant his team (9) and Shuster and Lubben (41). The issue of relocation and its consequences was also addressed in the Danish study (46), which found that the majority of transferred staff felt it was imposed on them and, although they had received training, they did not feel adequately prepared. The analysis both identified sources of potential conflict between staff and revealed deeper problems inherent in the strictly hierarchical structure of the medical professions. Relations between nurses and doctors require more in-depth analysis, as the pandemic has brought to light old tensions that have not yet been adequately addressed.

### Work stress

The last question related to the respondents’ and their colleagues’ assessment of their commitment to task performance during the pandemic. Medical performance depends on teamwork, which in turn influences perceived work stress. Therefore, we were interested in how respondents perceived their own and their colleagues’ engagement in task completion during a pandemic. We also wanted to compare work stress before and during the pandemic.

Respondents in our study rated their commitment slightly higher than that of their colleagues (mean 4.9 and 4.4 respectively), but overall the rating was high. This may be related to the importance our respondents attach to their profession. Organizational commitment can have a positive impact on the effectiveness of work and it can improve the perception of quality of life (47).

Unexpected situations usually lead to changes in routines and can lead to an increase in work stress. Therefore, we asked our participants about their assessment of stress levels before and during the pandemic. We found that the average self-assessment of work stress increased from 3.7 to 5.1 on a scale of 1 to 6. Being a frontline worker means a huge responsibility and anxiety at the same time. Interestingly, our respondents were more concerned about the transmission of the virus to their relatives than about their own infection. It is worth highlighting the fact that they were afraid of making a medical mistake because they lacked the appropriate competences or were exhausted. These findings suggest the need for adequate procedures and support for the physical and mental health of healthcare professionals. Increased stress levels are reported in almost all studies mentioned in this article. Our analyses point to possible causes for this and therefore make it possible to address specific solutions.

In addition, the in-depth interviews shed further light on the potentially stressful situations. The interviewees emphasised that they were dealing with a completely new type of patient - usually unconscious and connected to a ventilator. Even if they are conscious, communication with them is difficult because of the PPE worn by the medical staff. The patients are isolated and lonely and many of them die without the assistance of their relatives, making such situations emotionally difficult for medical staff. All this increases stress levels, which can have a negative impact on the mental health and physical condition of medical staff, which has been described in detail in the extensive literature (7, 13, 33, 35, 37, 38, 48–52).

## Study limitations

The first limitation is that the study was conducted under pandemic constraints, which narrowed down the choice of research methods and target population. Since we used an online survey, we had limited influence on the participants who completed the questionnaire.

Secondly, the response rate was moderate. Due to the restrictions, we could not directly ask medical staff to participate in the study. Limited access to medical facilities made it difficult to conduct the study and forced us to resort to indirect communication.

Third, our results cannot be generalised to a wider population. They only reflect the opinions of those who agreed to participate in the study.

Telephone interviews did not allow us to observe respondents in terms of their reactions and emotions that accompanied them during the interviews, so we rely only on the verbal communication channel.

Snowball sampling could influence the structure of the sample in terms of the psychological profiles of our respondents. It is possible that people who are vulnerable to being influenced by others and who have certain experiences and more radical opinions participated in the study. However, since we cannot determine whether our suspicions are confirmed in reality, we are inclined to consider this as a limitation of the study.

Another limitation is the structure of the participants in terms of their professions and place of work, which did not allow us to gain a deeper insight into the differences between their experiences and opinions depending on their professional situation. The sample contained an overrepresentation of nurses, followed by doctors and midwives. Other medical professions were only sporadically represented in the study.

## Conclusion

The study conducted contributes to existing literature by describing the extent of changes in the work of health workers during the first phase of the SARS-CoV-2 pandemic in Poland. It also highlights the main challenges that healthcare workers faced with the outbreak of the new virus and how this affected their relationships with colleagues and patients. In addition, the study looks at health workers’ greatest fears and shows how their stress levels increased during the pandemic. These issues have hardly been addressed in the Polish literature so far, so we consider our study a valuable source of information. Although many improvements have been introduced since the SARS-CoV-2 outbreak, the long-term effects of work overload, relocation and working under stress on the mental and physical health of healthcare professionals in Poland should remain a matter of concern.

Our findings suggest that lack of human resources seems to be a major problem during the pandemic. Without trained staff, even the highest quality medical equipment cannot save patients’ lives.

In addition, our study has identified sources of potential conflict between doctors and nurses, especially in critical situations, and it has uncovered a deeper problem that lies in the strict hierarchical structure of the medical professions. The relationship between nurses and doctors thus needs to be analysed in more detail, as the pandemic has brought to light old tensions that have not been adequately addressed so far.

Another conclusion from the study is the need for adequate procedures and support for the physical and mental health of medical staff. In almost all the studies mentioned above, increased stress levels are reported. Our study identifies possible causes for this and therefore makes it possible to address tailored solutions.

Finally, our findings allow for major conclusions to be drawn for the organisation of the health care system in the event of another pandemic. These relate in particular to organisational and personnel issues. Professional training in the operation of modern medical equipment and the performance of medical procedures should be offered to the vast majority of medical staff, not only to those already working in the ICU. Hospitals should employ coordinators who are responsible for keeping track and responding immediately when a problem arises. The areas of responsibility of the individual medical professions should be clarified and worked out in detail to avoid interprofessional conflicts.

## Data availability statement

The original contributions presented in the study are included in the article/Supplementary material, further inquiries can be directed to the corresponding author.

## Ethics statement

Ethical review and approval was not required for the study on human participants in accordance with the local legislation and institutional requirements. The patients/participants provided their written informed consent to participate in this study.

## Author contributions

PP, MW, and AP-K designed the questionnaire and interview questions, and edited and approved the initial version of the manuscript. EG and WC-A revised and accepted both research tools, provided interviewees for in-depth interviews, and revised and approved the final version of the manuscript. PP, MW, AP-K, EG, and WC-A collected the quantitative data. PP and AP-K conducted interviews and prepared transcripts, and performed the statistical analyses. PP prepared descriptive analyses and wrote the original draft of the manuscript. MW conducted the literature search and analyses and discussed the results. All authors contributed to the article and approved the submitted version.

## Conflict of interest

The authors declare that the research was conducted in the absence of any commercial or financial relationships that could be construed as a potential conflict of interest.

## Publisher’s note

All claims expressed in this article are solely those of the authors and do not necessarily represent those of their affiliated organizations, or those of the publisher, the editors and the reviewers. Any product that may be evaluated in this article, or claim that may be made by its manufacturer, is not guaranteed or endorsed by the publisher.

## Abbreviations

SARS-CoV-2: severe acute respiratory syndrome coronavirus 2
COVID-19: coronavirus disease 2019
HCW(s): heath care worker(s)
WHO: World Health Organization
PPE: personal protective equipment
ICU: intensive care unit
SARS: severe acute respiratory syndrome
MERS: Middle-East respiratory syndrome
I_1,2,3,4,5,6,7,8: subsequent interviewees in the qualitative study
NS: not significant
